# Navigating intersecting identities and stigmas - narratives from women who drop in and out of PMTCT care in western Kenya

**DOI:** 10.1186/s12913-026-15271-x

**Published:** 2026-11-05

**Authors:** Katrine de Angeles, Anna Kågesten, Morten Skovdal, Anna Mia Ekström, John Dusabe-Richards, Eunice Kaguiri, Ruby Killingsworth, Edwin Were, Elisabeth Faxelid

**Affiliations:** 1 Department of Global Public Health, Karolinska Institutet, Tomtebodavägen 18B, Stockholm, SE-171 77 Sweden; 2 Department of Public Health, University of Copenhagen, Copenhagen, Denmark; 3 Department of Clinical Science and Education, Karolinska Institutet, Södersjukhuset, Stockholm, Sweden; 4 Department of Infectious Diseases/Venhälsan, Södersjukhuset, Stockholm, Sweden; 5 Partners in Prevention, Moi University, Eldoret, Kenya; 6 Department of Reproductive Health, Moi University School of Medicine, Eldoret, Kenya

**Keywords:** HIV, Kenya, PMTCT, Intersectionality, Care engagement, Sexual and reproductive health

## Abstract

In resource-limited settings, defaulter tracing is a key strategy for re-engaging women living with HIV and their infants who miss scheduled clinic visits for the prevention of mother-to-child transmission of HIV (PMTCT). This qualitative study seeks to understand the social realities of women in western Kenya who drop in and out of PMTCT care despite continuous tracing efforts to keep them in care. Fifteen in-depth interviews were conducted, with ten women traced and re-engaged in routine care. The data were analysed via a narrative approach and interpreted and discussed through an intersectional lens. The findings are presented in two constructed type narratives, illustrating PMTCT care (dis)engagement as a complex and social process and practice that involves navigating multiple intersecting identities and stigmas. Gender power imbalances and poverty, together with intersectional stigma, needs, and dependencies, stood out as obstacles to sustained engagement. Food security and caretaking of loved ones took precedence over scheduled clinic visits, despite the appreciation of defaulter tracing and social support from health care providers. We call for the recognition of how the gendered and complex lived realities of many women in resource constrained contexts, in different ways and to different degrees, intersect with their PMTCT care engagement and challenge equitable HIV care and prevention.

**Supplementary Information:**

The online version contains supplementary material available at https://doi.org/10.1186/s12913-026-15271-x.

## Introduction

When it’s time to go to the clinic and there is no fare for transport and we haven’t had any food, what do you do? My husband also says that I am not supposed to go anywhere or get a job because I need to care for the children.

Despite decades of efforts to end the global HIV pandemic, women (cisgender) remain disproportionately affected by HIV [1], particularly in sub-Saharan Africa (SSA) [1, 2]. In 2022, 52% of new HIV diagnoses in SSA occurred among women of reproductive age (15–49 years), compared with 27% of their male counterparts [1]. Beyond biological factors, structural, social, behavioural, and cultural factors – especially gendered norms that affect women’s agency and ability to make decisions about their health, finances, and contraceptive use – also influence HIV outcomes [1, 2]. According to the United Nations Population Fund (UNFPA), only half (47%) of married women aged 15–49 years in Eastern and Southern Africa report being able to make their own decisions about their sexual and reproductive health, a key indicator for Sustainable Development Goal 5 on gender equality [3].

Care programs that prevent vertical transmission of HIV, commonly referred to as mother-to-child transmission (PMTCT), specifically aim to reach pregnant women with HIV up to two years postpartum to ensure their own health and that of their infants, as well as secondary prevention of HIV transmission to any sexual partners. Treatment failure is thus the key concern for women in PMTCT care who miss their scheduled clinic visits [4]. The standard PMTCT care continuum, adapted in line with country procedures, includes scheduled clinic visits from monthly to every 3–6 months and viral load monitoring of the woman every six months [4]. Starting and retaining pregnant women with HIV on antiretroviral treatment (ART) is critical for effective PMTCT and to ensure the health and wellbeing of both women and their infants. The WHO Option B+ policy, recommending lifelong ART for pregnant and breastfeeding women living with HIV regardless of CD4 count, has consequently been a gamechanger for ending vertical transmission [4]. However, nearly one-third of all new HIV infections in children in Eastern and Southern Africa occur because women drop out of PMTCT care [1] and loss-to-follow-up (LTFU) is a major concern in countries with high rates of vertical transmission [5]. LTFU calls for innovative approaches to engage women in care, which is why immediate tracing via telephone calls or home visits to re-engage women in PMTCT is initiated when women miss their scheduled visits [4].

PMTCT care engagement is challenging in various ways. Quantitative data [6–10] and interviews with health care providers (HCPs) [11] and women adherent to PMTCT care [12] identified disengagement factors as either biomedical (side effects) or behavioural and socio-structural (stigma, lack of social support, negative HCP attitudes, unemployment, poverty, poor knowledge of HIV, late initiation or being newly diagnosed, having poor mental health, intimate partner violence (IPV) and being of younger age) [9, 12–16]. A recent Kenyan study revealed that being late for scheduled clinic visits might even serve as an indicator of becoming LTFU [7]. Conversely, counselling, having good knowledge of PMTCT, higher education, social support, partner support, disclosure [6, 7], and male involvement have all been found to protect against PMTCT drop out [15].

Social and gendered roles, identities, and dynamics are at the core of several of these disengagement factors, yet we know little about how they unfold among women who drop out of, or with cyclical engagement in PMTCT care. A study from Kenya found that women who were LTFU also experienced diminished agency and expressed fear of partner abandonment and abuse, as well as fear of stigma related to their poor economic status [14]. This is consistent with findings from Zimbabwe [17] and Tanzania [18, 19], where women who were LTFU were found to experience emotional stress and difficulties coping with the diagnosis, IPV, and abandonment upon disclosure to their partners. The studies from Tanzania further discussed how stigma and either fear of or lack of social and partner support led to decisions of non-disclosure and treatment abandonment due to IPV or poverty [18, 19]. Concerns of isolation, lack of support from family or friends, and partner separation were equally raised among a cohort of pregnant women in PMTCT in Kenya [16].

Like many other East African countries, Kenya continues to face challenges related to structural gender inequalities. Only 39% of married women of reproductive age reported that they could make decisions about their health, according to the 2014 Kenyan Demographic and Health Survey [20]. Women from western Kenya, where our study was conducted, are less likely to have a say in overall decision-making than women in other Kenyan regions [20]. Acknowledging that gender inequality may influence HIV care, and in commitment to the UNAIDS 95-95-95 target, Kenya’s recent HIV care and treatment guidelines now include a patient- and family-centred approach and measures to address and screen for mental health challenges and gender-based violence (GBV), including violence from intimate partners [21]. Defaulter tracing for PMTCT care was introduced in Kenya in 2012 and routinely used to re-engage women who miss scheduled clinic visits via phone calls, home visits and community outreach [21]. Women who are traced are offered enhanced adherence support to help stay engaged in care [21].

## An intersectional lens

Intersectionality seeks to understand how various social identities and other categories of difference intersect in creating privileges or disadvantages [2, 22]. Social identities can be understood as the positions individuals occupy within social structures (i.e. marital status, gender, income group, age) and are often tied to or expressed through social roles (i.e. mother, wife, occupation, youth), which carry culturally defined expectations and responsibilities. In the exploration of intragroup differences [23], intersectionality seeks to understand how dynamics and systems of power intersect and operate at multiple social and ecological levels (individual, interpersonal, organisational, and social) to both drive and sustain inequality [2, 24]. This means that individuals’ life experiences, opportunities, and constraints are influenced by the hierarchical systems of power inherent in their social identities [22, 25].

Stigma, which refers to the devaluation of groups or individuals based on identities or roles seen as socially undesirable (i.e. HIV status, low-income, single/divorced) [26], is deeply entrenched in these power dynamics. It exists in multiple forms; perceived (communal), enacted (own prior experience), anticipated (expectations of stigma), or internalised (self-depreciation), all of which may be intersectional and multi-directional [26]. In this paper, we will refer to these constructs (social identities, roles, stigmas) as *‘intersections’* when addressed collectively.

Intersectional inequality for people living with HIV shapes everything from initial risk to exposure, the experience of living with HIV, medical and programmatic responses [2, 29] to care-seeking behaviour and linkage to care [27]. Women living with HIV who have children carry multiple intersections, that may influence their ability to access and engage in care [28–30]. In the pursuit of gender-equitable HIV care and prevention, recent publications call for expanding research on intersectionality and intersectional stigma beyond high-income countries [31, 32], particularly through qualitative research with those directly affected. Studies on HIV prevention and care in SSA have used intersectionality to understand the experiences of sexual and gender minorities [33, 34], female sex workers [28], adolescents [35], and people with disability [36]. Still, to our knowledge, no study has applied intersectionality to explore the experiences of women enrolled in PMTCT care specifically.

Echoing the call by Ma et al. 2016 [37], to explore how intersecting social identities, roles and stigmas may be reinforced by structural factors that affect care engagement, this study applies an intersectional lens to narratives that portray the lived experiences of women who drop in and out of PMTCT care in western Kenya despite continuous tracing efforts. By using a narrative analysis approach, we aim to illustrate the complexity of women’s social realities and explore how this group of women navigate PMTCT care (dis)engagement amid structural inequalities and layered stigma. We further aim to identify missing pieces to how policy and practice may be more effectively tailored to person-centred care.

## Methods

### Study design and setting

We conducted a qualitative study exploring care engagement for PMTCT as part of the broader WelTel-PMTCT trial, which took place between 2015 and 2019 in western Kenya. Described elsewhere [38], WelTel-PMTCT was a multicentre, two-armed randomised controlled trial to evaluate the effect of weekly interactive text messages to improve retention in care among women enrolled in PMTCT at six healthcare facilities. Additional data from this trial [39] explored the implementation of the national defaulter tracing policy through interviews with PMTCT HCPs and management at the county level.

For the present qualitative study, data collection was nested in the control arm of the trial at five healthcare facilities across three western Kenya counties: one sub-county hospital, a sub-district hospital, a county referral hospital and one national referral hospital catering to both urban and peri-urban populations. At the time of data collection, the HIV prevalence in these counties ranged between 5% and 17% [40], with the HIV prevalence among cisgender women being 60% higher than among men in two of the three Kenyan counties included [41].

PMTCT care provision at the participating facilities followed the national Kenyan guidelines (Option B+) from 2016 [41]. It was offered through the implementing partner, the Academic Model Providing Access to Health Care (AMPATH), in cooperation with the Ministry of Health. At the time of the study, PMTCT care was also integrated into regular maternal and child health clinics at the participating facilities, and mother‒infant dyads were seen together regardless of HIV status. A range of support structures was available at the facilities, such as mentor mothers (peer mothers trained to offer psychosocial support to other women living with HIV), defaulter tracing, facility- and community-based support groups, assisted disclosure, male-involvement, and support for vulnerable and/orphaned individuals [42]. Women who missed their scheduled visit were categorised as “missed visit”; after one week of absence, they were considered a “defaulter”; and after three months of absence, they were considered LTFU. Defaulter tracing via phone calls (after 24 h of absence) and home visits (after seven days) was performed by facility outreach teams consisting of community outreach retention workers, and community health volunteers who came from the facility catchment area [41].

### Participants and sampling

Participants in the current study were women enrolled in PMTCT care at the five facilities, who were recruited via purposive sampling to capture what facility staff would refer to as either “notorious defaulters” or so-called “No Show” mothers, known to show up late for their scheduled visits. To be eligible for an in-depth interview (IDI), women had to (1) be ≥ 18 years of age; (2) be enrolled in PMTCT care at a participating facility during the time of recruitment; (3) have at least three separate rounds of phone calls and/or a home visit initiated through the PMTCT defaulter tracing mechanism, a criterion developed together with the PMTCT facility staff members; and (4) have returned to PMTCT care after tracing. After an initial screening to exclude women classified as LTFU, transferred out, or with adverse outcomes or faulty IDs, eligible women would be approached by facility PMTCT staff via phone calls (the majority) or in person at their scheduled clinic visits to ask for their participation in an IDI and one round of follow-up IDIs. A total of 16 women expressed interest in participating, 10 of whom took part in at least one interview. Five of the ten women participated in a follow-up interview despite several attempts to reach all participants, for a total of 15 interviews.

### Procedures

The interviews were conducted when women were between 11 and 24 months postpartum (average 18 months). Four interviews were conducted in September 2017, with two follow-up interviews in March 2018 and six conducted in February/March 2018, with three follow-up interviews in July 2018. We kept 4–6 months between the initial and follow-up interviews to explore how the lives and experiences of our participants would unfold over the later postpartum period (10–24 months after birth), a period where women are known to be vulnerable to attrition [1, 5]. The follow-up interviews aimed to advance our understanding of participants’ everyday lives, contexts, and vulnerabilities that could elicit care disengagement. The interviews were audio recorded, lasted, on average, 36 min (ranging from 23 min to 1.5 h), and were conducted either at the study office or in a private space inside or outside the facilities according to the participants’ preferences. 150 Kenyan shillings (≈ 1.37 USD commensurate with local prices) were reimbursed to cover transport costs.

The first author (KDA), an experienced qualitative researcher and cisgender woman of Latin American origin in her late twenties, performed all the interviews. Twelve interviews were conducted in English, one in an English/Kiswahili mix, and two in Kiswahili, with assistance from a male research assistant as an interpreter. Neither of the two were known to the participants before the interview, but they were familiar with the PMTCT study setting, the research assistant through years of active social work at one of the facilities, and KDA through spending > six months in the research setting over a two-year period. KDA is considered an outsider, and the research assistant both an outsider and insider; he came from the same community and worked at one of the facilities, but is an outsider as a cisgender male.

The interviews were conducted using an interview guide (see supplementary file) designed by authors KDA, EK, EF, AM, MS and the Kenyan research assistants, informed by findings from prior literature. The guide was structured to solicit narratives in five areas related to care engagement: (1) PMTCT care, (2) defaulter tracing, (3) re-engagement in care, (4) life situation, and (5) living with HIV. The questions were open-ended and adapted iteratively as the conversation unfolded. Analytical memos [43] were written promptly after each interview. Follow-up interview guides were tailored to each participant based on insights from the initial interview, identifying themes and areas requiring further exploration. Prior to the follow-up interviews, KDA read the transcripts and listened to the audios to prepare herself. Each interview would start with participant validation, asking them to elaborate or clarify information from the previous interview when needed. Sociodemographic data were captured through questionnaires administered through the WelTel trial. The study adhered to the Consolidated Criteria for Reporting Qualitative Research (COREQ) guidelines [44].

### Data analysis

The interviews were transcribed verbatim and, where needed, translated into English. Quality checks were performed by the interviewer (and interpreter) promptly after transcription.

We applied a narrative analysis approach and interpreted our constructed narratives through an intersectional lens to provide contextualised insights into the dynamic ways women navigated everyday life and engaged in PMTCT care. Narratives were chosen for their ability to portray holistic accounts of lived experience, integrating both context and themes to capture how lives are constructed through interactions with the surrounding social environment [45, 46]. Our analytical process was done in an iterative and compound approach of connecting and categorising data [47] initially as ‘narrative finders’ and later as ‘narrative creators’ [48]. First, KDA and the senior author (EF) re-read all transcripts several times to familiarise themselves with the content and identify emerging stories. Second, in exploring how participants position themselves within their stories, we were attentive to social contexts, relationships, beliefs, and attitudes [49], and drew on holistic content analysis by Lieblich et al. [50] to identify emerging stories, themes, and patterns. Story segments specifically related to PMTCT care engagement were then coded inductively, from which interconnected themes emerged (sharing, support system, competing social roles, agency, stigma). These themes were organised into a coding matrix to enable comparison across interviews and to explore for similarities and differences. Themes and story segments were then linked and interpreted in relation to one another. This process led to the development of separate, restructured, and chronological narratives for each participant, primarily composed of verbatim interview data. These narratives provided a longitudinal account of living with HIV and navigating care engagement alongside multiple social roles, identities and stigmas.

Guided by Dewey’s notion that a person is understood only in interaction with others [51], we then used our matrix and the three-dimensional space narrative approach: interaction (personal and social), continuity (past, present, future), and situation (place) [49] to group similar narratives together. This step in the analysis resulted in two constructed type narratives [48] reflecting the lived experiences of two archetypes of women traced in PMTCT care in our study: the *young single mother* and the *married mother.* These narratives illustrate how participants navigated living with HIV, intersecting social roles, identities and stigmas, as well as and the factors that facilitated or challenged their care engagement.

Finally, we applied an intersectional lens to our narratives to interpret and discuss how women’s social contexts, power imbalances, and stigmas associated with their social identities and roles influenced PMTCT care (dis)engagement. This final step of analysis was triangulated with Kenyan co-authors (EK, EW) for analytical rigor and enhanced credibility.

### Ethics

The study was approved by the Institutional Research and Ethics Committee at Moi University, School of Medicine, Eldoret, Kenya (FAN; IREC 1292), and the Regional Ethics Review Board, Stockholm, Sweden (2018/742 − 31/1). The participants (above the age of 18) provided oral and written consent and were informed that they could withdraw from the study at any time. The study had supporting measurements to follow up on any clinical or psychosocial issues that could arise during the interviews. All participants are referred to by pseudonyms to protect their identities.

## Results

Ten women participated in this study, most of them were married (8/10), had disclosed their HIV status to a current spouse or former partner (9/10), and were housewives/casual labourers (8/10); only two were formally employed. Three had primary education only, four had secondary education, and three had vocational/tertiary education. With the exception of one woman, all reported contracting HIV due to infidelity from their sexual partner. Seven women received a home visit through defaulter tracing from their facility’s outreach team. However, one of them had only consented to follow-up via phone calls, highlighting a mismatch between the tracing method and her stated preference. In total, eight out of ten participating women had consented to home visits (see complete characteristics in Table 1).

**Table 1:** Characteristics of study participants

Pseudonym	Age	Nr. of Children	Marital status	Occupation	Education	Year diagnosed HIV	Mode of HIV acquisition	Disclosed to	Method traced	Tracing preference (Home visit or call)
Achieng	38	2	Married	Employed	Tertiary/Vocational	2004	Husband	Husband, sister, boss	Home visit	Both
Miriam	25	1	Single	Employed	Tertiary/Vocational	2010	Former boyfriend	Sister, mother, friends, father of child	Home visit	Both
Lensa	27	1	Single	Student/ Casual labour	Tertiary/Vocational	2015	Former boyfriend	Mother, siblings, friend	Home visit	Call
Mary	28	3	Married	Housewife (want to work)	Primary education	2014	Husband	Husband, sister, father in law, mother uncle, friend	Phone calls	Both
Naliaka	37	5	Married	Housewife (want to work)	Secondary education	2016	Husband	Husband, pastor, 3 sisters	Home visit	Both
Eunice	21	2 (and 3 orphans)	Married	Housewife (want to work)	Secondary education	2014	Husband	Husband	Home visit	Both
Cherotich	31	3	Married	Casual labour	Secondary education	2009	Former husband	Former and new husband, mother	Phone calls	Call
Eve	30	3	Married	Casual labour	Primary education	2010	Husband	Husband, friend, parents	Home visit	Both
Nemeye	27	2	Married	Housewife (want to work)	Secondary education	2016	Former boyfriend	Husband	Phone calls	Both
Carole	25	2	Married	Casual labour	Primary education	2012	Husband	Husband, mother, sister	Home visit	Both

The two ensuing type narratives, the married mother and young, single mother, are constructed based on the lived experiences of the 10 interviewed women who, despite numerous tracing efforts, dropped in and out of PMTCT care in western Kenya. These narratives, using primarily verbatim data extracts, illustrate PMTCT care (dis)engagement as a complex social process and practice shaped by structural conditions, interpersonal relationships, and intersections. In the sections that follow we present each constructed narrative, subsequently interpreted through an intersectional lens. Finally, we compare and contrast elements from the two narratives as either care-engagement facilitators or care-engagement barriers.

### Navigating care engagement for PMTCT: the married mother

This narrative is modelled based on interviews with Nemeye (25), Carole (31) and Mary (28) and constructed based on their lived experiences, with elements from interviews with five other married women (see Table 1). It captures how married mothers living with HIV in our study engaged with and disengaged from PMTCT care over time.

*“Before discovering my HIV-positive status, my health was deteriorating, and I struggled to conceive. I confided in a friend who advised me to get tested. The news devastated me, and I cried while the nurse compassionately counselled me to accept my status. I felt despair and feared telling my husband. With assistance from nurses, both my husband and I tested positive days later at the clinic. We began blaming each other, but I knew of his drinking and infidelity. He even refused condoms and was probably aware of his status. My brother, who had paid to take me through secondary school, was injured in an accident, so I only ever married to support myself because life became hard.*

*The HIV diagnosis, despite taking months to accept fully, sparked a journey of self-reflection. Initially, I felt worthless and lost weight, and even if I was already a mother of one, I considered throwing the medicine away for good and ending my life. But I discovered that I was pregnant again, and through an encouraging clinic nurse, I learned that I could have an HIV-negative child. It changed everything. I decided to take the ARVs not to offer my children a motherless life on the streets. Life with my husband was not good, and I lived in constant fear, but I started leaving the house again and gradually found happiness, realising that a strong heart can conquer any obstacle. My mom was a big inspiration in this, raising eight children alone. We are close, but I never disclosed to her, only my sister, and she offers financial and emotional support when able.*

*The joint support from these women gave me courage to divorce my hurtful husband. Now, at 31, I am happily married to a supportive and encouraging husband who reminds me of clinic appointments and to embrace life as it comes. We are blessed with three children, two from my previous marriage and one from my current husband. All HIV-negative like him. Although we face financial instability and live in a rental home, I strive to be strong for my family. In support of my husband hawing in the markets, I occasionally wash and sell fruits and vegetables when it’s season. It challenges my clinic attendance, but going to the clinic is less of a priority when your children go to bed with an empty stomach, so I grab most opportunities for casual labour. I also travel to see relatives and care for my children and husband when they are sick. The nurses reprimanded me in the past and called me careless when I missed visits, making me reluctant to come. However, after sharing my life story with them, they showed me compassion, and we connected. They no longer judge and know I am steadfast in taking my medication even if I do not always show on time. Nowadays, a nurse neighbour can even bring my medicine when I lack far to go to clinic. To have warm-hearted neighbours uplift me and give me strength, too.*

*Once, I travelled to visit my in-laws with the children for a month, missed my appointments, but continued my ARVs. Then, two healthcare workers came to my home to check on me. Despite expressing my preference for calls or messages to avoid potential gossip or embarrassing my husband, I warmly welcomed them and appreciated their caring approach. They respected our privacy, were kind to the children, and emphasised the importance of regular clinic attendance. Now, I call the clinic if I cannot make it, and they call to remind me of my appointments, too.*

*Today, I am healthy and grateful for this transformational journey. I prioritise my well-being, aim to become a mentor mother and share my story of hope and acceptance with those who feel helpless. I want to provide the same unity and connection that made me feel valued and motivated at the clinic. The lessons learned that a woman with a job is independent and strong and that staying active relieves the mind from stress. My heart now finds purpose in life’s challenges, and I trust God’s guidance.”*

### An intersectional lens: married mother

The married mother’s care engagement was shaped by intersections involving her HIV diagnosis, gender, marital status, income level, motherhood, and mental health, as well as competing interests and needs. Some of her social roles carried structural limitations and cultural norms, which then led her to prioritise caregiving and food security over scheduled clinic visits. Intersecting stigma equally challenged her care engagement compounded by other forms of marginalisation, including poverty and gender inequality.

Internalised HIV-related stigma initially led to denial and suicidal ideation. These emotions were subsequently reinforced by enacted stigma from her former husband and poor attitudes from some HCPs. Together with IPV and poverty, these intersecting factors negatively impacted her mental health and her motivation to engage in care. However, systems of social support from her sister, the new husband, neighbours and non-judgemental HCPs facilitated care engagement and self-acceptance. Sharing her struggles with them provided emotional relief and encouragement for self-transformation, acceptance, and liberation. Over time, several stigmas were dismantled in this relational and internal process; her care engagement improved as she established autonomy from harmful relationships and received affirming feedback from health workers, illustrating both the severity of PMTCT care barriers related to intersections, and the ways in which these barriers can be overcome.

### Navigating care engagement for PMTCT: young, single mother

This narrative is modelled and constructed based on interviews with Lensa (27), supplemented with experiences from interviews with Miriam (25) and two married women who had prior experience going through PMTCT as young single mothers (see Table 1).

*“I didn’t feel like something was wrong with my body, so maybe God alerted me. I had little faith in my boyfriend, and as a joke, I went for testing with my cousin. When I learned my positive status, I truly feared; it was not my expectation, but I accepted and started medicines one week later. The doctor said I could live longer that way.*

*I called my mother the same day; she comforted me to continue living like before, to focus on my studies and God’s purpose for me. My brothers and sisters also know, not my dad, he is a lion and very harsh. I even took him to the sub-chief when he refused to take me to college. My boyfriend denied having HIV, so I ended the relationship. Maybe he knew his status and did it to me intentionally. It was hurtful, but I don’t look down on myself. The doctor told me to stop drinking, only have one boyfriend or girlfriend, and use protection because I am positive, but I wanted a break from all the directives, and when I joined college, I ended up pregnant with a college mate. He disappeared, and I hid the pregnancy from my family, fearing their judgment and disappointment. I isolated myself and sometimes slept hungry. My parents eventually found out. They asked me to come home and said the mistake [child] could be a lastborn. It was a stressful and emotional time for me, knowing also that a teacher from school was banned from the institution because she was single and pregnant.*

*Financially, things are difficult. My dad used to pay my rent before he got sick, and my mom had no money. I ask my family for food but feel like another burden for them. Sometimes I skip school and clinic to wash people’s clothes to pay rent. I am lucky that my older sister takes care of my daughter while I finish my diploma in agriculture and that my daughter tested negative for HIV because. My sister was not diligent with her prophylaxis, and I came to breastfeed my daughter until she was one year even while she is living with my sister.*

*My situation makes it challenging to manage my clinic appointments. At my place, I might miss clinic, and I travel home a lot, usually for a month at a time, and I can’t always know how long I will be away. Sometimes fare is a problem, my mum is sick, or I must stay and help. The staff at the clinic have become my friends, and the head nurse is like a mother to me. I trust that they do not disclose my private information. I gave them my contacts, mum, and sisters – in case I don’t show up. I pray they only find my mom if they show up at our family home; better if they go to my place where it’s just me. So far, they have called but several times, and the more they call, the more I feel an urge to come. Sometimes they quarrel me, and I promise to come via SMS. I never call, never tell them why I am away. I show up when I am able because I am embarrassed. I have private numbers, and if they are not there when I go for clinic, I can also stay away. Last time when I travelled, I only brought Septrin [antibiotic to prevent opportunistic infections]. They could have given me extra medicines and postponed my date if I had notified them.*

*I am advised to go to the nearest clinic when I travel, but most people there are relatives; even at the pharmacy, they call me Aunty. Villagers spread gossip, and if my dad finds out, he will imagine all the money he spent and think: “We are burying this one tomorrow”. For this reason, I don’t like to carry drugs when I travel. It’s a tough dilemma. I don’t like missing clinic, and I know I am risking my life. I had friends who unfriended me because they suspected my status, and I have seen friends sick that I buried. They stopped their medicines, but I am here and need my health so I can benefit from my education when I graduate.*

*These experiences changed my view on relationships and self-sufficiency. I will not need a man if I get a job after graduation. My daughter gives me the company I need; my priorities are her well-being, education, and a life of freedom. One day, I will ask anyone for water to take my ARVs and inspire others to live with pride and determination, single mothers too. If we were open, we could help one another instead of destroying each other with secrecy.”*

### An intersectional lens: young single mother

The young single mother was similarly challenged by the intersections of living with HIV as a young, unmarried, single mother with low socioeconomic status. She strived for food-security, independence, self-acceptance and a better life, while navigating the expectations and norms of her social roles, as a student, daughter, mother, and a patient. This narrative further illustrates how intersectional stigma undermined her care continuity and led to social isolation.

She faced both internalised, and enacted stigma related to HIV, poverty and a pregnancy outside of marriage at a young age from her educational institution: and she feared perceived and anticipated judgement from her father, extended family and her community.

These fears discouraged travel with ARVs and hindered regular clinic attendance, despite her awareness of the associated risks. While social support from her mother and HCPs inspired her to embrace herself and help others, her fears, social dependencies, limited agency and ability to cope with stressors challenged these aspirations.

### Care-engagement barriers

The above narratives display the composite and interwoven ways in which overlapping social identities, roles, and stigmas shaped care disengagement for the women who participated in our research. In both narratives, women described how they navigated multiple intersections within constrained structural conditions, including poverty, gendered power imbalances, and limited autonomy. They weighed viral suppression, physical clinic attendance, and their own needs and desires, against those of loved ones, HCPs, educational institutions, and workplaces, and allocated time and energy, considering the demands and expectations of all parties in their lives. In both cases, food insecurity and competing domestic or income-generating responsibilities, such as caretaking of loved ones and ensuring daily survival, took precedence over scheduled clinic visits.

The narratives reveal vast differences in women’s social realities and the impact of their intersections. Married women initially refused their ARVs, struggling with internalised stigma exacerbated by longstanding IPV and gender subordination. Their HIV diagnosis was deeply entwined with their marital dynamics, which initially included male dominance and enacted stigma from their partner, challenging their mental wellbeing and beliefs that they could have a good and financially secure life living with HIV. Young single women, by contrast, encountered multiple stigmas associated with gender, age, pregnancy outside of marriage, often compounded by the stress of coping with a recent HIV diagnosis. While young single women did initiate ARVs immediately, many of their decisions were constrained by fear of disclosure and public ridicule, especially when travelling to rural areas, where familial and communal ties were stronger. Their limited access to financial resources and weaker social safety nets, overall, made them more vulnerable to disengage from care. All women in our study expressed fear of social exclusion.

Gender power imbalances manifested in everything from women’s ability to negotiate monogamy and condom use to financial constraints. Married women initially relied financially on their brothers and later their husbands, while young single women were dependant on their fathers. Both groups faced ongoing financial constraints and restricted access to income-generating activities; for married women these constrained were compounded by lower educational attainment.

While women described how defaulter tracing was appreciated and served as a reminder, the acceptability and accessibility of care remained a barrier considering the prevailing structural conditions and stigmas that the women faced.

### Care-engagement facilitators

Both narratives also highlight how women’s engagement in care was facilitated through multi-level social support and processes of self-reflection and transformation. Women described how emotional and practical support from HCPs, family members, peers and religiosity, motivated them to actively (re)engage in care. Sharing their concerns and sorrows, appeared to have created a shift in women’s personal beliefs, enabling them to reframe their experiences of HIV, motherhood, and womanhood. Across both narratives, these types of social interaction also increased women’s knowledge about living with HIV. This internal shift manifested in new ways of taking responsibility for their lives, including making deliberate choices to increase independence from men and financial independence. The ability to translate social support into self-empowerment, and leave limiting beliefs and attitudes behind, however, varied between the women.

Narratives from married women illustrate growing confidence, increased agency, and resistance to internalised stigma, which are facilitated by systems of support from new partners, family members, and HCPs. The passage of time, repeated counselling, and motherhood (including the desire to prevent transmission to their children) similarly catalysed this shift in self-perception, strengthening their motivation to remain in care and abandon suicidal ideation. Conversely, single women, despite expressing a desire to ensure their own and their children’s health and well-being, demonstrated a lower sense of agency, and struggled to assert their needs amid multiple dependencies and limited psychosocial support. Although they valued support from HCPs and family, it was not sufficient to offset the effects of their unstable living conditions, and the intersecting stigmas they faced.

Before sharing their struggles, both groups of women expressed a lack of understanding from their HCPs, but it did not keep them from attending care.

Overall, our analysis suggests that intersectional stigma, when compounded by other intersecting structural and relational vulnerabilities, undermines women’s agency and ability to engage in care. Social support and time to adjust may disrupt these dynamics, particularly when women have access to nurturing care relationships and the opportunity to reclaim their decision-making power through community and enabling care facilities.

## Discussion

We set out to explore the social realit​ies and experiences of women who, despite numerous tracing efforts, drop in and out of PMTCT care in western Kenya. We have presented narratives from a vulnerable sub-group of women at risk of dropping out of PMTCT care owing to their lived realities and failure to consistently attend scheduled clinic visits. Intersections, gender power imbalances, and facilities’ inability to accommodate the complex social realities of women stood out as key findings. While noting overall similarities, our study points to significant differences in how these factors shape PMTCT care (dis)engagement for married and young single women, which is discussed further below.

### Intersections

Our inductive, narrative approach revealed how the accumulation and interaction of unequal gender powered relations, social identities and roles, poverty, and multiple forms of stigmas collectively shaped women’s engagement in PMTCT care. These findings led us to move beyond the socioecological model commonly used in prior PMTCT care engagement research [14, 52, 53], adopting instead an intersectional lens to delve deeper into the relational and structural dynamics that seemingly influenced PMTCT care (dis)engagement for women in our study. Unlike previous studies, women did not speak of side effects [19], long waiting queues [13], poor HCP attitudes [13] or religiosity [14] as reasons to miss their scheduled clinic visits. Poor HCP attitudes were mentioned as a stressor for women in our study, but they did not prevent them from attending care. Moreover, consistent with previous studies [7, 13], we noted apparent differences by age and marital status; Our findings question the use of fragmented and isolated factors to understand women’s care engagement behaviours. Rather than individual identity markers, it was intersections, rooted in power and structural inequity, that co-shaped women’s (dis)engagement with PMTCT care, as exemplified by the distinct experiences of married and young single women.

In particular, the intersection between living with HIV and being a mother played a dual role in both facilitating and challenging care engagement. Initially, married women refused ARVs because of stress from IPV and the acceptance of living with HIV. However, pregnancy awakened a newfound desire to prioritise their health and safeguard their unborn child, corroborating previous findings from Kenya, South Africa and Tanzania [54–56]. Despite this, married women prioritised more immediate caregiving responsibilities following motherhood over scheduled clinic visits. While these actions may reflect internalised maternal norms, their narratives emphasised individual motivations rather than communal expectations and societal norms of being “a good mother”, as observed in other Kenyan studies [28, 57]. By contrast, we found that social norms related to motherhood were a significant challenge for young single mothers in our study. Initially, they concealed their pregnancies but later navigated motherhood by relying on family members to balance school attendance with childcare responsibilities and ensure food security for themselves and their children. Unfortunately, inconsistent provision of infant prophylaxis by family members meant knowingly putting their child at risk of vertical transmission.

These findings underscore previous findings from Kenya and South Africa of women as deliberate decision-makers regarding their PMTCT care engagement, strategically navigating and shifting identities in trying to conform with societal practices, expectations, and stigmas [28, 55, 57–59].

Notably, while all the women in our study disclosed their HIV status to their husband or (former) partner – mirroring the high disclosure rates (73% − 95%) reported in previous studies [14, 16, 60], they still struggled to navigate PMTCT care. This may be partly because many, in particular, young, single women, had not disclosed their status to other family members and the broader community, emphasising how disclosure mediates the level of support that women in PMTCT care can access [1]. Akin to previous studies from SSA [12, 13, 16, 61], all women in our study feared or experienced social abandonment and exclusion owing to harmful gender norms and experienced or internalised stigma because of their HIV-positive status or pregnancy. In our study, young single women refrained from taking their ARVs when travelling due to fear of social exclusion. The intersectional stigma, anticipated, perceived, experienced, and internalised, faced by young single women in our study from their HIV status, having a child outside of marriage, and younger age may exacerbate this fear and contribute to nondisclosure. This corroborates findings from a recent western Kenyan study noting how nondisclosure of one’s HIV status limits women’s ability to navigate and integrate their intersecting identities into their daily lives while in PMTCT care [61, 62].

### Gender power imbalances

Experiences of IPV were common among the married women in our study; these experiences are associated with seropositivity, treatment nonadherence and LTFU [63] and are known to affect women’s mental well-being and ability to cope with living with HIV [13]. When married and single women initially disclosed their status to their male partners, it led to increased emotional violence [56, 62] and a lack of partner support [13, 61]. This finding underscores the need for PMTCT care to foster openness and for research to explore the use and effectiveness of existing IPV and GBV screening programs within PMTCT care in SSA.

Contrary to findings from Uganda and Malawi, where women felt incomplete without a male partner and stayed in harmful relationships that damaged their care engagement [61], the married women in our study demonstrated agency. Encouraged and inspired by PMTCT providers, they divorced and found a supportive husband. The young single mothers in our study similarly chose to stay single rather than depend on a male partner. Despite this, the women in our study lived in poverty, and the majority were homemakers without higher education or stable income-generating activities, reflecting the general profile of women in the larger WelTel PMTCT trial [16]. This meant that they still relied primarily on male family members to make ends meet, either a father or a new husband, which made them vulnerable in managing their HIV diagnosis.

Our findings illustrate how gender power imbalances act as prevailing barriers to PMTCT care engagement [1, 14]. They further represent gendered dimensions of vulnerability affecting women and children in general in low- and middle-income countries [64] and underscore male involvement as crucial to improving PMTCT care engagement [65]. This should be accomplished via a systems approach [66] involving couples counselling and testing [62], community sensitisation and assisted disclosure [1], male-friendly PMTCT services, and assessments of behaviour both inside and outside the home and across the PMTCT care continuum [67].

### Complex social realities

Attending scheduled clinic visits was desirable for the women in our study, but their complex social realities limited care engagement. Having a linear care continuum and expecting complex realities to conform to the existing care setup might not be realistic for this subgroup of mothers in PMTCT who are negotiating their health and well-being alongside poverty, stigma and harmful gender norms. This finding aligns with that of Lazarus et al. (2023) [68], who underscore that the management of HIV demands attention to the whole person to succeed. This includes addressing medical, psychological, social, and economic aspects of people’s lives and the practical and symbolic layers that inhabit them [2].

Since our study, policy shifts globally [1] and in Kenya [21] have made strides towards person-centred PMTCT care, which holistically considers factors that affect the continuity of care. As such, Kenya updated its HIV policy in 2022 to include individualised adherence support plans for all people living with HIV [21]. This was followed by an action plan for PMTCT released in late 2023 that conversely raised concerns about disparities and insufficiencies in implementation of PMTCT policies [69]. Thus, implementation research is needed to explore the progress, implications, and outcomes of policies for women in PMTCT care programs in Kenya. In optimising care programs, our findings highlight a dire need to understand intergroup differences among women who continuously disengage and re-engage in care in large-scale studies, as well as desires and levels of involvement from their male partners. Our findings provide HCPs and policymakers with valuable insights and add to the evidence base of how PMTCT care engagement for young single women and married women is shaped differently. We suggest that our findings can assist HCPs and managers in implementing PMTCT policy and in determining how to approach these segments of women to enable person-centred care throughout the care continuum. For person-centred care solutions to succeed, we propose structural combination interventions at community-level using participatory designs and sensitisation. We also concur with Tourneau et al. (2022) and urge further research into optimised delivery and monitoring strategies [70] for these subgroups of women, including the practical possibilities and implications of distributing ARVs closer to women. Narratives in our study also reflect expressions of individual and collective agency. We propose future research to integrate intersectionality with collective agency theory [71] to better understand how collective agency may disrupt and dismantle intersectional stigma and facilitate women’s sustained engagement in PMTCT care. Taken together, our findings echo the 2023 UNAIDS recommendations and the newly released Kenyan Action Plan to end AIDS in Children [69], which address both IPV and gender norms and stress the importance of including women in shaping PMTCT policy and care in alignment with their unique needs and lived realities.

### Strengths and limitations

While the narratives in the present study are not exhaustive, they make a valuable contribution to research into why women circle in and out of PMTCT care. To our knowledge, no other study has interviewed women with HIV about their PMTCT tracing experience and used an intersectional lens to understand their care engagement dynamics. While this study is part of a larger study exploring the implementation of the Kenyan defaulter tracing program for PMTCT, the sample size for this sub-study is small which may limit the transferability of our findings. The narratives reflect the data from 10 women in PMTCT care enrolled in the WelTel trial. This excludes stories from women who were illiterate, < 18, without access to cell phones or LTFUs. As a hard-to-reach population, it was difficult to recruit women for the study; one-third of all those who agreed to participate never showed up, half never came for their scheduled re-interview, and only two participants were single mothers at the time of the interview. Similar studies, nonetheless, report similar challenges with a median of only 18 participants [14, 17–19], and it is possible that women with experience of stigma and discrimination from their facilities and HCPs refrained from participation. That the study was conducted at high volume clinics in urban/peri-urban areas, may similarly limit transferability to more remote clinics with less infrastructure or less resourced implementing partners. The fact that the first author spent extensive time at the facilities and communities and discussed findings with research assistants and Kenyan co-authors however helped to increase credibility and transferability, along with member checking from participants who were reinterviewed. The average interview length in this study was 36 min, yet we believe that our data held sufficient information power [72] to adequately address our research question. But, to have probed participants further about desires for male involvement in their PMTCT care would have enriched our findings.

Further studies are needed on the desire for male involvement among this subgroup of PMTCT mothers. Our data were collected from 2017 to 2018. Kenya updated its HIV policy in 2022, adding additional support to people living with HIV, including individualised adherence support plans. Further research is needed to explore whether this policy change has improved care engagement for women in PMTCT who cycle in and out of care or become LTFUs.

## Conclusions

The linearity of the PMTCT care continuum fails to accommodate the complex social realities of women with HIV, who strategically prioritise more immediate needs over scheduled clinic visits while navigating intersecting identities, roles and stigmas. Living with HIV is but another intersecting social identity that challenges the health and overall well-being of this subgroup of mothers who continuously disengage and re-engage in PMTCT care. These dynamics shape care engagement differently for married women and young single women and hamper equitable HIV care and prevention.

Tracing women in PMTCT may be appreciated and serve as a care reminder but does not address the greater socio-structural challenges that leave women vulnerable, dependent, and at risk of social exclusion. We call for the recognition of how the gendered and complex lived realities of many women in poor resource contexts, in different ways and to different degrees, intersect with PMTCT care engagement.

## Supplementary Information

Below is the link to the electronic supplementary material.

Supplementary Material 1

## Data Availability

The datasets used for the current study are available from the corresponding author on reasonable request.
