# Supplementary material for: Navigating intersecting identities and stigmas - narratives from women who drop in and out of PMTCT care in western Kenya
Source: BMC Health Serv Res. 2026 Nov 5;26:1544. doi: 10.1186/s12913-026-15271-x (PMC13741409; doi:10.1186/s12913-026-15271-x)
Supplement: Supplementary file 1 [file 12913_2026_15271_MOESM1_ESM.pdf]

WelTel PMTCT Qualitative defaulter tracing Study –

Narrative Interview Guide – Traced Patients in control arm

ID #: _______________ Date of interview: _____________ (dd-mm-yyyy)

Interviewer's initials: ____________________ Location of interview: ____________________

OP / No /: ____________________________ Type of informant________________________

Length of interview__________________

Section 1: Introduction

Thank you for taking your time to talk with me today. We are conducting this interview with you to listen to your experience receiving PMTCT care, and your opinions on successes of and barriers to being retained in care and your experience with being contacted if you missed a PMTCT event. You have been invited to participate in this interview because you are a PMTCT client at one of the participating clinics. At anytime, you are free to stop the interview if you wish to or let me know if you do not wish to answer a specific question. The study is being conducted under the direction of Professors Anna Mia Ekström and Edwin Were.

I will now start asking you some questions. When you answer I would like you to imagine that you are telling me the story of your PMTCT experiences. There are no right or wrong answers, and you are free to tell me anything you want to share with me. Do you have any questions before we start?

Section 2: Assess PMTCT clients´ experience with PMTCT care

1. What is it like for you to be in PMTCT care and what are the stories from your experiences in coming to care from when you started care to now?

Probes: Who did you talk to? What did you feel? What happened next? What did you do? Why did you choose to…? How did you remember to? Why did you forget to…? Who were you with? Are you still in the same clinic?

Specifying: Can you give me examples?

Direct: When you mention, do you mean…?

Section 3: Assess PMTC clients' experiences and perceptions of PMTCT patient defaulter tracing

3. Have you been called or visited because you missed a PMTCT appointment?

Probes: Do you have more than one experience? Where you called or did you experience a home visit? How many times where you called/visited?

4. Could you please tell me the story of what happened when you were called because you missed a scheduled visit?

Probes: Why did you miss? Who contacted you? What visit(s) did you miss? How did you feel when you received the phone call? What did the person tell you? How did you feel about what you were told? What happened next?

5. Could you please tell me the story of what happened when you were visited in your home because you missed a scheduled visit?

Probes: what happened when you were visited in your home? How did you feel? Where were you when you were visited? Who was with you? What happened? Who said? What happened next? Where you called before? Why did you choose to?

Section 4: Assess PMTCT clients´ experience of coming back to PMTCT care

6. Can you please tell me about coming back to care?

Probes: What happened? Why did you decide to? Was it something someone said? Something that happened in your life? Why did it make you return? How did you feel? What happened next?

Direct: When you mention, do you mean…?

Section 5: Asses PMTCT clients' experience of being back in PMCT care

7. What is it like for you to be back in PMTCT care?

Probes: How do you feel? Who? Why did you?

Direct: When you mention, do you mean…?

Section 6: Assess the life situation of the PMTCT client

8. Can you please tell me about your life situation, what is your life like?

Probes: Who do you? How did you experience? How do you feel? What is it like to? Where do you?

Specifying: Can you give me an example of a typical day?

Section 7: Asses PMTC clients' experience with HIV

9. May I ask you, what it has been like for you to live with HIV?

Probes: How did you initially feel? When was? How do you feel now? Do you think about it? Who? How have people around you responded? How do you experience? What did you know about HIV before?

Section 8: Closing

10. Thank you for sharing your stories with me. Do you have anything else you would like to share that you feel is important but we have not talked about?

Is there anything you would like to add or change?

END

Comments about the context (filled by interviewer)**:**
